# Margin derivation from intrafraction patient motion of multi‐target, single isocentre, brain stereotactic radiosurgery treatments

**DOI:** 10.1002/acm2.14405

**Published:** 2024-10-18

**Authors:** Misael Caloz, Sébastien Tran, Max Gau, Edouard Romano, Nikolaos Koutsouvelis, Pelagia G. Tsoutsou

**Affiliations:** ^1^ Department of Radiation Oncology Geneva University Hospital Geneva Switzerland; ^2^ Faculty of Medecine University of Geneva Geneva Switzerland

**Keywords:** margins, mono‐isocentric radiosurgery, radiotherapy, surface monitoring

## Abstract

**Background:**

Brain metastases are the most common intracranial malignancy and remain a substantial source of morbidity and mortality in cancer patients. Linear accelerator based stereotactic radiosurgery (SRS) is widely used and is frequently delivered by hypo‐fractionnated volumetric modulated arc therapy using non‐coplanar beams, where geometric accuracy and planning margins are a major concern.

**Purpose:**

To give a practical analysis of intrafraction patient motion for multi‐target, single isocentre, brain SRS treatments and to derive adapted GTV‐to‐PTV margins.

**Methods:**

Data of 154 lesions, spread over 85 fractions from 56 patients treated in our institution with the Varian HyperArc  SRS solution was processed. Intrafraction patient motion were recorded using an Optical Surface Monitoring System during irradiation. The present study focuses on small tumor volumes, roughly equal or inferior to 1.5 ${\mathrm{cm}}^{3}$, and frameless mask‐based immobilization. For each treatment session, a tumor displacement vector matrix was calculated from the patient drifts as a function of time. Data were combined together into a representative treatment scenario and the dosimetric impact of GTV displacement was calculated.

**Results:**

Recommended margins due to patient motion range between 0.3 and 1 mm, depending on the distance tumor‐isocentre, and the desired GTV edge dose coverage. Those values should be added quadratically with other sources of uncertainty, such as mechanical isocentre and kV‐MV misalignment.

**Conclusion:**

Thorough analysis of intrafraction patient motion was performed, the dosimetric impact was calculated for different scenarios, and adequate GTV‐to‐PTV margins were derived. These values vary according to the distance isocentre‐to‐GTV, as well as the desired dose coverage, and should be chosen adequately.

## 1 INTRODUCTION

Brain metastases are the most common intracranial malignancy and remain a substantial source of morbidity and mortality in cancer patients.^1^ Incidence of brain metastases has risen as cancer treatments have improved and patients with metastatic disease live longer.^2^, ^3^ Linear accelerator (linac) based stereotactic radiosurgery (SRS) is widely used for the treatment of brain metastases and intracranial primitive lesions. Radiation dose is frequently delivered by volumetric modulated arc therapy (VMAT) using non‐coplanar beams, depending on the linac's technical capabilities. In single‐fraction or fractionated radiosurgery, geometric accuracy as well as planning margins are always a major concern. Thus an accurate patient positioning and a stable immobilization are essential to reach high‐accuracy dose delivery. Historically, invasive head frames have been considered robust and accurate, although frameless masks can reach comparable accuracies with appropriate image guidance.^4^ Nowadays, frameless masks are commonly used in clinical practice for SRS treatment, usually in combination with kilo‐volt (kV) and/or mega‐volt (MV) imaging.

To date, few studies have been published on setup uncertainties and margins of such technique. Tsuruta et al.^5^ studied intrafraction patient motion with the ExacTrac x‐ray system (ETX, Brainlab AG, Olof‐Palme‐Straße 9, 81829 Munich, Germany). In a similar approach with the ETX system, Eder et al.^6^ studied the patient misalignment for non‐coplanar beams and its dosimetric impact on the target coverage. Duan et al.^7^ focused on the setup errors and margins on a anthropomorphic phantom. Interestingly, Zhang et al.^8^ reported their results on setup uncertainties obtained with ETX measurements and derived corresponding margins for both invasive head ring and non‐invasive thermoplastic masks. However, their margins derivation come from setup error (systematic error), and not from intrafraction patient motion (random error). Besides, it only concerns single target treatments.

Up to this date, there is no clear consensus on the margins to apply to the Gross Tumor Volume (GTV) of multi‐target, single isocentre, brain SRS treatments when using frameless masks. The present work aims to give a practical analysis of clinical data and to derive adequate margins from intrafraction patient motion, using surface monitoring technology. In this study, only the drifts from intrafraction patient motion are considered.

## 2 MATERIALS AND METHODS

### 2.1 Patient data and HyperArc

**FIGURE 1:**
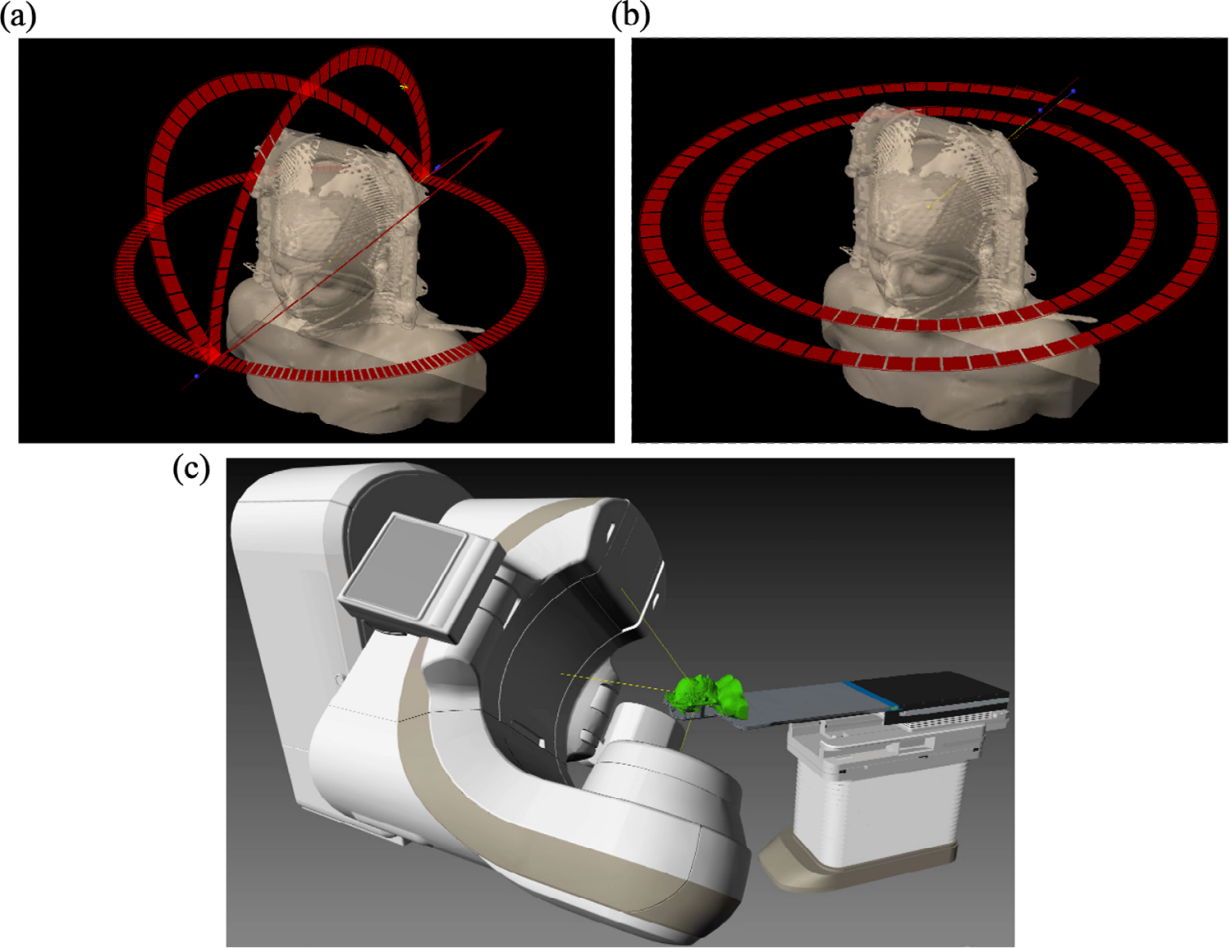
(a) Non‐coplanar arcs as used in HyperArc  treatments versus (b) conventional coplanar arc configuration. (c) Schematic of gantry and table rotations necessary to set up non‐coplanar beams.

The present study retrospectively analyzed all the patients treated in our institution between 2019 and 2022 that underwent HyperArc  treatments. This is the widespread Varian (Varian Medical Systems, Inc. 3100 Hansen Way, Palo Alto, California, USA) solution for brain SRS that comprises specific immobilization masks (Qfix Encompass SRS Fibreplast systems, CQ Medical, 440 Church Rd Avondale, PA 19311, USA), collimator and gantry angles, table rotations, and treatment planning software (TPS) optimizer, see Figure 1. The inclusion criteria were: immobilization with the above‐mentioned frameless masks; single‐fraction or hypo‐fractionation brain SRS treatments; surface monitoring data available during the entire treatment. They were included regardless of tumor's topography, location, and number. A total of 154 lesions and 85 fractions over 56 patients were included for analysis. Tumor‐to‐isocentre distances ranged between 0 to 62 mm.

### 2.2 Treatment planning and delivery

GTV and organs at risk (OAR) were delineated by physicians following current SRS guidelines,^9^, ^10^ with Varian Eclipse software. The doses were prescribed to the 80% isodose line, meaning the 100% isodose covers the edges of the PTV and the dose maximum is capped to 125% of the prescribed dose at the center of the GTV. Margins of 1 or 2 mm were added to the GTV, depending on the target‐isocentre distance, and target size. The doses were optimized and calculated using the Varian HyperArc module, where three to five coplanar and non‐coplanar beams were set‐up, depending on the complexity of the optimization and treatment plan. The dose was delivered by a Varian TrueBeam linac, with 120 leaves high‐definition (HD) multi‐leaf collimator (MLC).

### 2.3 Surface monitoring

**FIGURE 2:**
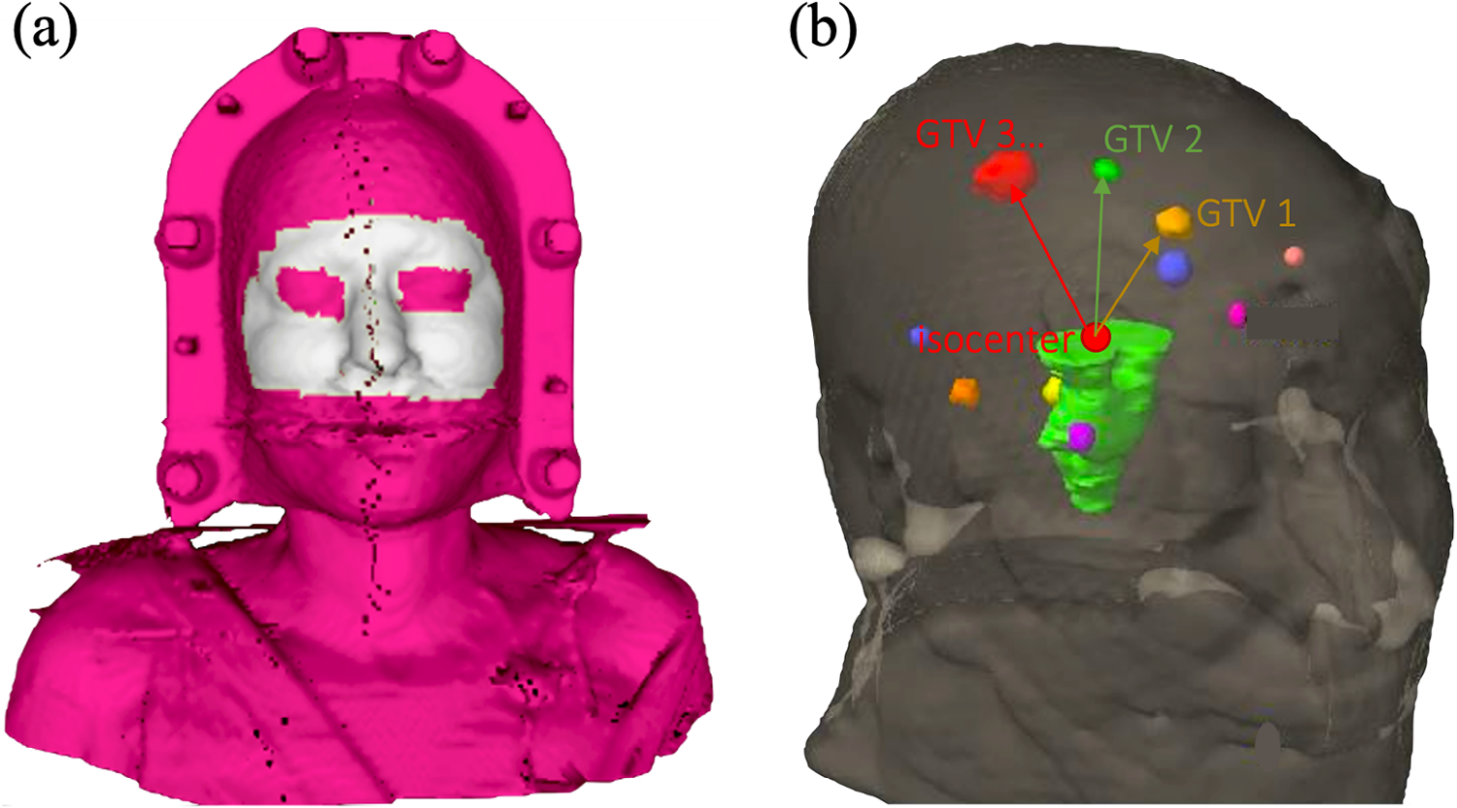
(a) AlignRT surface ROI example, shown in grey. (b) Example of a 3D schematic of brain multi‐metastasis, the isocentre is placed automatically at the centroid of the GTVs grouping. GTV, Gross Tumor Volume; ROI, region of interest.

All brain SRS treatments were monitored in real‐time with AlignRT, an optical surface monitoring system (OSMS) from Vision RT (Vision RT Ltd. Dove House Arcadia Avenue, London N3 2JU, UK), which offers sub‐millimeter monitoring accuracy. More specifically, this system consists of three cameras installed on the ceiling of the treatment room, that shine a light pattern at a specific wavelength on the patient surface, and record the back‐scattered light. The frameless masks are open around the nose and the eyes, allowing accurate measurement directly on the skin, as shown in Figure 2a. Due to the non‐flat patient surface, the back‐scattered geometric pattern is modified, and the surface is 3D‐modelized using calculation algorithms.

The algorithm transforms (translation and rotation) the surface extracted from the dosimetric plan (what we call the *reference surface*) to match the *measured surface* (surface as measured by the system during irradiation). The system reports in real‐time three translational and three rotational drift values in its coordinate system, which define the deviation of the *measured isocentre*, compared to the *reference isocentre* (which is extracted from dose plan). The drifts are: vertical; lateral; longitudinal; yaw; pitch; and roll. In our treatment conditions, the uncertainties of the surface monitoring system were evaluated to be ± 0.2 mm.^11^, ^12^, ^13^ More specifically, this uncertainty is defined as the maximum measured error of a one‐shot absolute position measurement compared to the cone‐beam computed tomography (CBCT) ground truth (0.12 mm/0.04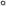 at 2.5 σ). In addition, in the present study the patient surface is constantly tracked and only the drift is measured, not the absolute position. In this case, the associated uncertainty is related to the measurement stability. AlignRT system measures approximately 15 times per second. With a refresh time of half a second (relatively small compared to the patient drifts), and assuming a normally distributed statistics, the uncertainty of the mean would be 0.04 mm (at 2.5 σ), which is by far smaller than any quantitative values derived in this paper. It must be noted that the reported drift are obtained by a rigid surface registration of the measured surface, compared to the reference surface.

### 2.4 Intrafraction motion data analysis

After patient positioning on the treatment table, appropriate alignment was performed with a CBCT at the beginning of each fraction, and MV images after each table rotation. Intrafraction position drifts were recorded as a function of time during irradiation. Data of all patients were combined together in the same data set without distinction, representing a typical treatment session.

We defined the *input vector* ${\vec{v}}_{i}$ that corresponds to the 3D imaginary coordinates of a GTV before applying the drifts, that is, the coordinates that would correspond to its position in the simulation CT. Similarly, the *output vector* ${\vec{v}}_{o}$ is defined as the 3D imaginary coordinates of the GTV after applying the drifts.

(1) $${\vec{v}}_{i}=\left(\begin{array}{c} x_{i}\\ y_{i}\\ z_{i} \end{array}\right)\;\mathrm{and}\;{\vec{v}}_{o}=\left(\begin{array}{c} x_{o}\\ y_{o}\\ z_{o} \end{array}\right)$$

The translational (${\vec{v}}_{T}$) and rotational ($M_{R}$) transformations were defined as

(2) $$\begin{array}{c} {\vec{v}}_{T}=\left(\begin{array}{c} \Delta x\\ \Delta y\\ \Delta z \end{array}\right)\;\mathrm{and}\;M_{R}=M_{\mathrm{yaw}}M_{\mathrm{pitch}}M_{\mathrm{roll}} \end{array}$$

with $M_{R}$ being a 3×3 matrix. $M_{\mathrm{yaw}}$, $M_{\mathrm{pitch}}$, and $M_{\mathrm{roll}}$ are the matrices corresponding to the rotational transformations, namely the yaw, pitch, and roll, respectively. More specifically, they are the usual 3D rotation matrices of Euclidean space and equal to:

$$\begin{array}{cc} M_{\mathrm{yaw}} & =\left[\begin{array}{ccc} cos(\theta ) & -sin(\theta ) & 0\\ sin(\theta ) & cos(\theta ) & 0\\ 0 & 0 & 1 \end{array}\right],\\ M_{\mathrm{pitch}} & =\left[\begin{array}{ccc} cos(\phi ) & 0 & sin(\phi )\\ 0 & 1 & 0\\ -sin(\phi ) & 0 & cos(\phi ) \end{array}\right],\\ M_{\mathrm{roll}} & =\left[\begin{array}{ccc} 1 & 0 & 0\\ 0 & cos(\psi ) & -sin(\psi )\\ 0 & sin(\psi ) & cos(\psi ) \end{array}\right] \end{array}$$

Applying the misalignment drift values to the input vector gives

(3) $${\vec{v}}_{o}={\vec{v}}_{T}+M_{R}\;{\vec{v}}_{i}$$

at any given time. For sake of readability, the dependence of time is omitted in the equations. But formerly, ${\vec{v}}_{o}$, ${\vec{v}}_{T}$, and $M_{R}$ depends on time. ${\vec{v}}_{i}$ does not depend on time, and can be arbitrary chosen at any point in space, in a way to map the 3D space of the brain. In our case, we chose to map uniformly input coordinates contained in a sphere of 6 cm of radius from the isocentre.

The distance from the input to the output coordinate, what we call the GTV *displacement* $∥{\vec{v}}_{o}-{\vec{v}}_{i}∥$, is obtained as a function of the input coordinate ${\vec{v}}_{i}$, at any given time.

Margins were finally derived in the following way: imaginary margins are added to a representative dose distribution around the GTV; we relate the dose received by the GTV border as a function of the intrafraction patient drifts, that way the GTV dose coverage can be calculated as a function of the margins. Finally, the recommended margins found depend on the GTV dose coverage desired, as well as on the distance GTV‐to‐isocentre.

### 2.5 Dose distribution

**FIGURE 3:**
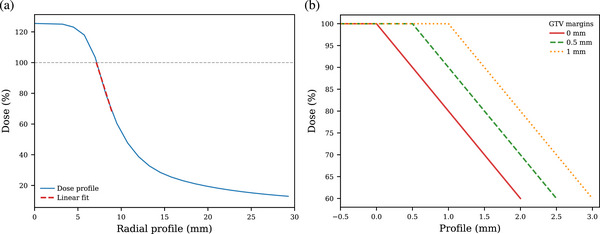
(a) Dose profile of a typical non‐coplanar treatment, calculated with the HyperArc module of the Eclipse treatment planning system. The red dashed line indicates the linear fit in the region of interest. (b) Dose profiles used in this study, as a function of different GTV‐to‐PTV margins. The position x=0 corresponds to the edge of the GTV, and negative values correspond to region inside the GTV. GTV, Gross Tumor Volume.

The typical dose gradient of a non‐coplanar treatment is shown in Figure 3a. For sake of calculation simplicity, the dose gradient is parameterized as a linear function, represented as a red dashed line. This simplification is valid in the region of interest, that is, where the drift values (indicated in Figure 5) do not exceed 2 mm. The GTV edge is never displaced to values above 2 mm, so that only the linear region of the dose profile can be used. It is assumed that the dose linear coefficient does not vary with the margins used, neither with the size of the target. This assumption has been verified for different target volumes. Dose profiles used in this study, as a function of different margins, are shown on the right plot of Figure 3. The dose inside the GTV is capped to 100%; this is mathematically necessary to ensure correct dose coverage normalization, as explained in the next section. In other words, dose higher than 100% of the prescribed dose that might occur in GTV hotspots does not compensate for lack of dose at the GTV edge.

**FIGURE 4:**
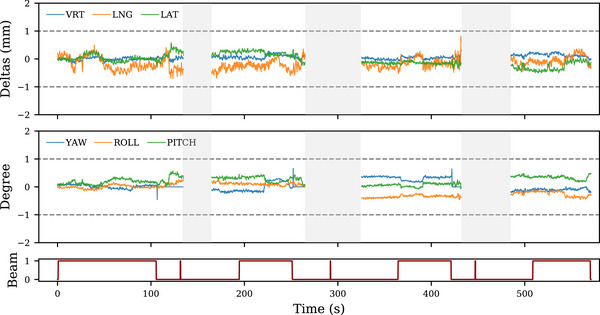
Data from a typical treatment session, showing the six drift values as measured by the OSMS, as a function of time. Bottom plot indicates the beam status, 1 for ON and 0 for OFF. In this particular example, four non‐coplanar beams were used. The three spikes in the beam status between arcs delivery correspond to MV‐imaging performed between each table rotation. Blank rectangles corresponds to specific gantry angles, in between arcs, for which the OSMS cameras were hidden and measurements could not be done accurately. MV, mega‐volt; OSMS, optical surface monitoring system.

**FIGURE 5:**
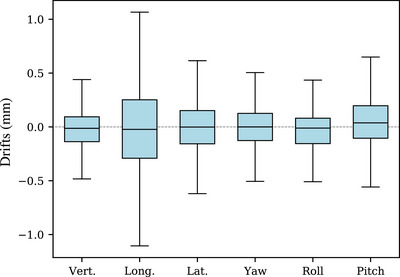
Box‐plot of the six drift values distribution measured by the OSMS, namely vertical, longitudinal, lateral, yaw, roll, pitch.

### 2.6 Margins derivation

**FIGURE 6:**
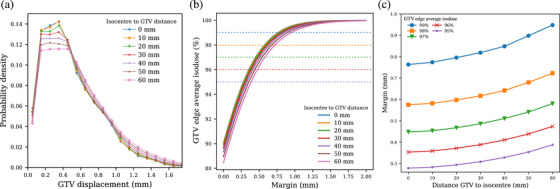
(a) Probability densities of the GTV displacement. Each color, associated to different markers, represents a different isocentre‐to‐GTV distance. (b) GTV edge average isodose as a function of the GTV margins, at different isocentre‐to‐GTV distance. The dashed lines represent specific dose levels. (c) GTV‐to‐PTV margins to apply as a function of the distance isocentre‐to‐GTV, to aim for a given GTV edge average isodose. GTV, Gross Tumor Volume.

As discussed in Section 2.4, the GTV displacement $∥{\vec{v}}_{o}-{\vec{v}}_{i}∥$ is obtained as a function of the input GTV coordinate ${\vec{v}}_{i}$. Displacements obtained from different ${\vec{v}}_{i}$ are regrouped depending on their distance from the isocentre, and combined into displacement histograms (Figure 6a). The displacement histograms are probability densities and their integral sum to unity, that is, the entirety of the treatment time is included in those histograms.

The probability density of the GTV edge isodose (${\mathrm{PDF}}_{\mathrm{edge}}$), that is, the lowest isodose line reached by a point on the GTV edge, is obtained by relating the GTV displacement probability density (${\mathrm{PDF}}_{\mathrm{disp}.}$, Figure 6a) to the parametrized dose distribution (Figure 3b). Mathematically, this corresponds to do the following variable substitution:

(4) $${\mathrm{GTV}}_{\mathrm{disp}.}\to \mathrm{GTV}\;\mathrm{edge}\;\mathrm{iso}\mathrm{dose}$$

In other words, for each GTV displacement values (from 0 to +∞, Figure 6a) we associate the corresponding isodose value (from Figure 3b). By doing so, we end‐up with the GTV edge isodose probability density ${\mathrm{PDF}}_{\mathrm{edge}}\left(D\right)$.

The GTV edge average isodose, that is, the average of the lowest isodose lines reached by a point on the GTV edge during a treatment fraction, is obtained from the following weighted integral:

(5) $$\begin{array}{ccc} & & \int_{0}^{+\infty }{\mathrm{PDF}}_{\mathrm{edge}}\left(D\right)\times D\;dD\\ & & =\mathrm{GTV}\;\mathrm{edge}\;\mathrm{average}\;\mathrm{iso}\mathrm{dose}\leq 100\%\;, \end{array}$$

with ${\mathrm{PDF}}_{\mathrm{edge}}\left(D\right)$ the probability density of the GTV edge isodose and D the isodose values as parametrized in Figure 3b. Note that from Equation 5, D should not be larger than 100% to ensure correct integral calculation. The GTV edge average isodose is then calculated with different margins and GTV‐isocentre distances using Equation (5).

In the process, we assumed that the dose is delivered uniformly in time during the treatment. This is statistically justified, since we analyze enough patient data, and that we only consider isotropic margins. This point is discussed in more details in Section 4.

Note that the GTV edge average isodose corresponds to the average of the lowest isodose lines reached by a point on the GTV edge during a treatment fraction, and that it quantifies the targeting mismatch of the worst case scenario. Additionally, the lack of dose at a specific GTV location may in principle be compensated later on by higher doses at that same location. While this may be valid in the perspective of radiobiology, additional geometric assumptions have to be made. For example, a completely randomly distributed drift values have to be assumed, which might not be the case. This detailed analysis is beyond the scope of this paper, and is left for future work.

## 3 RESULTS

### 3.1 Intrafraction patient motion

A typical treatment session performed in our institution is shown in Figure 4, where the six deltas are plotted as a function of time. As mentioned in Section 2, the drifts were recorded in real‐time during the entire session for all patients, and are combined together into a representative treatment session. The three translational drifts, vertical (SD = 0.24 mm), longitudinal (SD = 0.46 mm), and lateral (SD = 0.29 mm) are shown in box‐plots in Figure 5. Regarding the rotational drifts, results indicate the following: yaw (SD = 0.24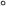), roll (SD = 0.20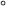), and pitch (SD = 0.28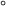).

Our treatment protocol defines thresholds of 1 mm and $1^{\circ }$ for translational and rotational drifts, respectively. This ensures high accuracy and positioning repeatability during treatments. Communication between the OSMS and the linac beam is not automatic, a radiation therapy technologist (RTT) is actively monitoring the patient motion drifts, and triggers a beam stop if one of the thresholds is exceeded. This explains why some longitudinal drift values shown in Figure 5 are above the defined threshold of 1 mm, as it corresponds to a small time delay of beam stops. This also ensures that no outliers are present in our data sets. Results of Figure 5 confirmed that 1 mm/$1^{\circ }$ thresholds are adapted.

### 3.2 Margins derivation

Following the methodology explained in Section 2, the GTV displacement $∥{\vec{v}}_{o}-{\vec{v}}_{i}∥$ is obtained as a function of the input GTV coordinate ${\vec{v}}_{i}$. Displacements obtained from different ${\vec{v}}_{i}$ are regrouped depending on their distance from the isocentre, and combined into displacement histograms, as shown in Figure 6a. Subsequently, the GTV edge average isodose is then calculated with different margins and GTV‐isocentre distances using Equation 5. The results are presented in Figure 6b and 6c and recommended margins are summarized in Table 1.

**TABLE 1:** Summary table of recommended margins due to intrafraction patient motion.

	Desired GTV edge average isodose		
Distance isocentre‐target	99%	98%	95%
Less than 20 mm	0.8 mm	0.6 mm	0.3 mm
Between 20 and 40 mm	0.85 mm	0.65 mm	0.35 mm
More than 40 mm	1.0 mm	0.7 mm	0.4 mm

*Note*: Mechanical isocentre and kV‐MV margins should be added quadratically with the chosen value.

## 4 DISCUSSION

Table 1 summarizes the recommended margins to be used to account for intrafraction patient motion. Note that those values should be added quadratically with other sources of uncertainty discussed in this section.

For SRS treatments, GTV‐to‐PTV margins are geometrical and isotropic margins to add to the GTV in order to cope with alignment errors and uncertainties. Those errors derive from different factors, such as: (i) intrafraction patient position drifts, as discussed in this study, (ii) machine mechanical isocentre misalignment, (iii) kV imaging system to MV isocentre misalignment. Undoubtedly, other factors contribute to the overall treatment accuracy, such as target delineation, image guidance, and MRI‐CT registration accuracy.^14^, ^15^, ^16^ The present study focused on intrafraction patient position drifts due to patient motion. On the contrary and by definition, the so‐called setup error, which is often considered systematic^17^ was not considered in this study, as this type of error is greatly reduced using appropriate image guided radiotherapy (IGRT) and 6D couches for SRS intracranial treatments.

Machine mechanical isocentre misalignment as well as kV‐to‐MV misalignment, despite being essential for the total margin derivation are not considered in this report and their thorough analysis will be the object of future work. Gao et al.^18^, ^19^ reports on off‐centre Winston‐Lutz measurements on a Varian TrueBeam linac and indicates a deviation ranging from 0.32 to 0.77 mm, depending on the distance from the isocentre. Interestingly, Wang et al.^20^ measured the kV‐to‐MV misalignment to be under 1 mm, also on a TrueBeam linac. However, for the two above‐mentioned studies, adequate margins remain to be defined. Note that margins are institution‐dependent and it is up to every single center to adapt them adequately to their own utilities.

Regarding other publications, a few studies have been published on setup uncertainties and margins. Some of them studied intrafraction patient motion without scrutinizing associated margins,^5^ while many others focused on dosimetric impact of a patient misalignment.^6^, ^21^, ^22^, ^23^ Only a few dived into margins derivation,^7^, ^8^ however Duan et al.^7^ concerns the setup errors and margins on a anthropomorphic phantom, while Zhang et al.^8^ reports on their margins derivation that come from setup error (systematic error), and not from intrafraction patient motion (random error). Up to this date, there is no clear consensus on the margins to apply to the GTV of multi‐target, single isocentre, brain SRS treatments when using frameless masks. The present work aims to give a practical analysis of clinical data and to derive adequate margins from intrafraction patient motion, using surface monitoring technology.

The calculated dose gradient has been verified not to change substantially for sphere‐shaped target volumes up to approximately 1.5 ${\mathrm{cm}}^{3}$ (corresponding to a sphere with a diameter of 1.42 cm). For larger volumes, additional care is necessary because of two reasons: the dose gradient may change, and the spherical symmetry (i.e., point transformations of Equation 3) assumed in this study would not be strictly valid anymore, and anisotropic margins should be derived.

As mentionned in Section 2, patients were immobilized using the frameless masks Qfix Encompass SRS Fibreplast system, which is widely used for brain SRS. Undoubtedly, the margins derived in this study directly depends on the type of frameless mask used. In addition, it also depends on the specific workflow followed in this work: MV‐images in‐between arcs with patient correction if necessary, constant surface monitoring, and 1 mm/1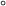 active threshold as explained in Section 3. Note that correction from MV‐images are done in the following way: if a shift is observed in the images, the table is set back to 0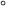 if it was not already, a CBCT is done, the related shifts are applied and the OSMS measured surface is reset to match the reference surface. Finally the table is rotated back to the treatment angle.

Even if it is possible to calculate it theoretically, the assumption that the dose is uniformly and symmetrically delivered in time is necessary in order to greatly simplify the calculations. Its validity is justified because it does not impact the final margins' derivations result. Essentially this simplification disregards the information of the exact point of dose‐coverage loss, which is blurred out around the border of the target volume. We consider these limitations acceptable, since only isotropic margins were considered.

## 5 CONCLUSION

An extensive analysis of intrafraction patient motion for multi‐target, single isocentre, brain SRS treatments was performed. For each treatment session, a tumor displacement vector matrix was calculated from the patient drifts as a function of time. Data were combined together into a representative treatment scenario and the dosimetric impact of GTV displacement was calculated. Recommended margins range between 0.3 and 1 mm, depending on the tumor‐isocentre distance, and the desired GTV dose coverage, as shown in Table 1. There values vary as function of the isocentre‐to‐GTV distance, as well as the desired dose coverage, and should be chosen adequately. Furthermore, uncertainties from different factors should also be taken into account, namely the machine mechanical isocentre misalignment as well as kV‐to‐MV misalignment. It relies on every individual institution to adapt their margins to their own utilities and take them into account into overall margins calculations.

## CONFLICT OF INTEREST STATEMENT

The authors have nothing to disclose, and there are no potential conflicts of interest.
